# Cognitive and Emotional Strain in Brain Tumor Patients: A cross-sectional study in a Resource-Limited Setting

**DOI:** 10.12669/pjms.41.13(PINS-NNOS).13445

**Published:** 2025-12

**Authors:** Momin Bashir, Muhammad Rohaan, Anam Arshed, Saad Raza, Omer Bin Adnan, Haseeb Mehmood Qadri

**Affiliations:** 1 Momin Bashir, Medical Student, Rutgers Robert Wood Johnson Medical School, NJ, USA; 2 Muhammad Rohaan, Medical Student, Aga Khan University, Karachi, Pakistan; 3 Anam Arshed, Medical Student, Rahbar Medical and Dental College, Lahore, Pakistan; 4 Saad Raza, Medical Student, Islamabad Medical and Dental College, Islamabad, Pakistan; 5 Omer Bin Adnan, Medical Student, King Edward Medical University, Lahore, Pakistan; 6 Haseeb Mehmood Qadri, MBBS, Punjab Institute of Neurosciences

**Keywords:** Brain Neoplasms, Mental Health, Resource-limited settings, Financial stress, Pakistan

## Abstract

**Objective:**

To evaluate the cognitive and emotional burden of benign and malignant brain tumors on patients in resource-limited settings to improve quality of life.

**Methodology:**

This prospective, cross-sectional study was conducted by the Punjab Institute of Neurosciences (PINS) in Lahore, Pakistan. Patients aged 18 years and older, diagnosed with at least one primary solid tumor and undergoing treatment at two major public hospitals in Lahore and Islamabad, were interviewed between March and April 2025 to assess their levels of mental strain and distress. The results were then analyzed descriptively.

**Results:**

The mean age of participants in this study was 36.72 years (SD = 15.74). Among the total sample, 62% (31) were male and 38% (19) were female. Tumors were benign in 66% (33) of cases and malignant in 34% (17). A majority, 56% (28), reported that the tumor had changed their outlook on life, while 16% (8) felt it somewhat changed, 24% (12) remained unchanged, and 4% (2) were unsure. For anxiety, 22% (11) reported daily feelings of nervousness and anxiety, while 30% (15) experienced daily worries. Concentration levels varied, with 50% (25) able to concentrate normally, 30% (15) having some trouble, 6% (3) unable to concentrate on anything, and 14% (7) unable to read, watch TV, or focus at all. In terms of financial burden, 48% (24) perceived a very high burden, 20% (10) felt it was somewhat high, 12% (6) reported a minimal burden, and 20% (10) experienced no burden.

**Conclusion:**

A brain tumor diagnosis presents significant challenges for patients, affecting them physically, mentally, and financially. In Pakistan, where mental health is often stigmatized, understanding these challenges becomes even more crucial. By addressing both the physical and mental health needs of patients, especially in resource-limited settings, targeted support can help patients cope more effectively with this devastating illness.

## INTRODUCTION

Cancer is one of the leading causes of death globally, responsible for nearly 1 in 6 deaths in 2020.^1^ Patients diagnosed with cancer often experience significant psychological distress, including anxiety and depression. These mental health challenges can complicate treatment, affecting both patients and healthcare providers.^1^

The global burden of cancer is growing, with low- and middle-income countries (LMICs), especially those with low- and middle-Human Development Indexes (HDIs), bearing a disproportionate share of this growth. Additionally, there are significant disparities between these countries and developed nations in terms of survival rates and the ability to prevent, detect, and treat cancer.^2^ This issue is especially relevant in Pakistan, where healthcare challenges are exacerbated. The country has seen an increase in cancer prevalence, likely driven by factors such as air pollution, smoking, dietary habits, and drug use.^3^ With one of the world’s lowest public health expenditures and among the highest out-of-pocket healthcare costs, approximately 85% of patients in Pakistan pay directly for their treatment. This financial burden significantly impacts patients’ psychological well-being, especially given the constrained access to government-run oncology centers and rehabilitation services.^4^

A brain tumor diagnosis, in particular, carries significant psychosocial risks, regardless of prognosis. Patients often experience psychological comorbidities, such as cognitive strain, adjustment disorders, and depression.^5^ Cognitive strain refers to a state of mental fatigue caused by prolonged cognitive effort, commonly seen in patients under psychological distress.^6^ It may present as impaired concentration, memory difficulties, reduced motivation, and subjective tiredness. Although not always accompanied by measurable performance decline, cognitive strain can still significantly affect patients’ daily functioning and quality of life. Compounding these challenges, mental health is heavily stigmatized in Pakistan and many other South Asian countries. It is rarely discussed or recognized as a serious threat to both mind and body. Patients with psychiatric symptoms in Pakistan are often marginalized, viewed as dangerous, and less likely to form social connections, which can lead to a decreased support network and poorer outcomes.^7^

There is a notable gap in research on the prevalence of psychological symptoms among cancer patients in Pakistan. Addressing this gap is crucial, given the rising risk factors and the minimal existing research. Doing so would improve the treatment of the psychological aspects of cancer, potentially leading to better overall outcomes. Additionally, it would help foster a broader dialogue on mental health in the country, challenging the stigma surrounding mental illness.

## METHODOLOGY

This prospective cross-sectional study was conducted by the Punjab Institute of Neurosciences (PINS), Lahore, Pakistan from March to April 2025. Non-probability, convenience sampling was employed. The minimum sample size of 31, corresponding to an 80% confidence level, was calculated using the OpenEpi sample size calculator, based on an estimated population of 2,750 brain tumor patients (as reported in a nationwide epidemiological study in Pakistan) and an anticipated depression frequency of 5%.^5^

### Inclusion Criteria:

Adults aged 18 or older with a primary solid tumor diagnosis who are undergoing active cancer treatment in two major public hospitals of Pakistan: PINS, Lahore, and the Pakistan Institute of Medical Sciences (PIMS), Islamabad.

### Exclusion Criteria:

Individuals under 18 years of age, those without a primary solid tumor, patients not undergoing treatment, individuals with cognitive impairments or unrelated psychiatric disorders, and those unable to provide informed consent were excluded from participation.

### Data Collection:

Data collectors were hired across the country on a voluntary basis through an application and interview process. They received specific training in conducting patient interviews and data collection methodologies to ensure consistency and reliability. Standardized, pre-validated questionnaires were selected, translated into Urdu (the only language understood by the patients), and then converted into a Google Form for use by the data collectors.

Prior to data collection, all participants were fully informed about the study’s purpose, procedures, potential risks, and benefits. Written informed consent was obtained from each participant to ensure that they understood their involvement and agreed to participate voluntarily. To protect participants’ privacy, all collected data was anonymized, and personal information was securely stored, accessible only to authorized research team members. Participants were also informed that they had the right to withdraw from the study at any time without any impact on their medical care. Although over one hundred patients were approached, only 50 met the eligibility criteria and agreed to participate in the survey.

During the interviews, each data collector filled out the Google Form questionnaire which also included an oath ensuring the anonymity of the survey responses. The oath also required patients to pledge that they would respond voluntarily, honestly, and according to their own interests. All interviews were recorded with the patient’s consent and were later verified by the study planners to ensure that the questions asked by the data collectors were consistent with the questionnaires.

### Mental Health Assessment Scales:

To capture both cognitive and emotional strain, the study employed multiple validated tools, each targeting specific dimensions of mental health. The Beck Depression Inventory (BDI) and Hospital Anxiety and Depression Scale (HADS) primarily assess emotional symptoms such as sadness, hopelessness, and anxiety.^8^,^9^ In contrast, the GAD-7 evaluates both cognitive and somatic symptoms of generalized anxiety, such as excessive worry and restlessness.^10^ Using multiple questionnaires allows for a more comprehensive and specific assessment of psychological distress. Each tool has its own diagnostic specificity: for example, GAD-7 has a specificity of ~0.82 for generalized anxiety disorder, while HADS-A and HADS-D are designed to minimize overlap with somatic symptoms, making them particularly suitable for medically ill populations.^8^,^10^

Because individual clinical diagnoses were not the goal of this study, standardized cut-off scores for tools like the HADS, GAD-7, or BDI were not applied. Instead, participants’ responses were analyzed based on frequency categories (e.g., “most of the time,” “occasionally”) to capture the overall emotional and cognitive burden across the sample. This approach allowed us to assess broader trends in psychological strain, rather than evaluating each participant’s clinical status individually. By aggregating response patterns, we aimed to gauge the collective mental health profile of the group, offering a larger view of distress within the sample without assigning diagnostic labels.

### Data Analysis:

Descriptive statistics were computed using Google Sheets to examine trends and patterns in the mental health status of the patients. The results were analyzed with a focus on the relationship between mental health and various demographic and clinical factors. This study was approved by the Institutional Review Board (IRB) of PINS via the reference number 2021/IRB/PINS/Approval/2025; Dated: January 23, 2025.

## RESULTS

The mean age of patients in this study was 36.72 years (SD = 15.74). Of the total sample, 62% (31) were male and 38% (19) female. Patients were treated at PINS/LGH, Lahore (76%, 38) and PIMS, Islamabad (24%, 12).

Regarding diagnoses, 28% (14) of patients had meningioma, 14% (7) had glioma, 10% (5) had pituitary adenoma, and 10% (5) had astrocytoma. Tumors were benign in 66% (33) of cases and malignant in 34% (17). Tumor locations were left-sided in 42% (21), right-sided in 40% (20), and midline in 18% (9).

In terms of patient understanding, 54% (27) reported a clear understanding of their tumor, and 22% (11) had a somewhat clear understanding. However, 20% (10) reported no understanding. Regarding life outlook, 56% (28) felt the diagnosis significantly changed their perspective, while 24% (12) reported no change. A large portion (84%, 42) expressed confidence in their treatment plan, with only 8% (4) uncertain (Table-I).

**Table-I:** The Patients’ Understanding of the Tumor.

Question	Percentage (Frequency)
How would you describe your understanding of the type of tumor you have?	• Very clear and complete understanding: 54% (27)
	• Somewhat clear understanding: 22% (11)
	• Limited understanding: 4% (2)
	• No understanding at all: 20% (10)
How do you feel about the tumor diagnosis?	• Very anxious or fearful: 40% (20)
	• Somewhat anxious or fearful: 22% (11)
	• Neutral: 18% (9)
	• Hopeful or positive: 8% (4)
	• Relieved or not concerned: 12% (6)
What emotions have you experienced since learning about your diagnosis? (Select all that apply)	• Anxiety: 28% (14)
	• Fear: 34% (17)
	• Sadness: 20% (10)
	• Confusion: 14% (7)
	• Anger: 14% (7)
	• Hope: 24% (12)
	• Acceptance: 24% (12)
	• None: 8% (4)
Do you feel that your tumor diagnosis has changed your outlook on life?	• Yes, significantly: 56% (28)
	• Yes, somewhat: 16% (8)
	• No change: 24% (12)
	• I am not sure: 4% (2)
Have you received adequate information about your tumor (e.g., size, location, and type) from your healthcare providers?	• Yes, very informative: 34% (17)
	• Yes, somewhat informative: 24% (12)
	• No, not enough information: 22% (11)
	• No, I did not receive any information: 20% (10)
Do you feel confident in the treatment plan proposed for your tumor?	• Very confident: 84% (42)
	• Somewhat confident: 8% (4)
	• Not confident: 6% (3)
	• I do not understand the treatment plan: 2% (1)
How much control do you feel you have over your health condition?	• A lot of control: 46% (23)
	• Some control: 34% (17)
	• Very little control: 10% (5)
	• No control at all: 10% (5)
Do you think having a benign or malignant tumor affects your emotional well• being differently?	• Yes, a benign tumor affects me differently: 30% (15)
	• Yes, a malignant tumor affects me differently: 42% (21)
	• No, both affect me similarly: 6% (3)
	• I am not sure: 22% (11)
What do you fear most regarding your tumor diagnosis?	• Fear of death: 24% (12)
	• Fear of disability or loss of function: 24% (12)
	• Fear of the treatment process: 4% (2)
	• Fear of the impact on family and relationships: 22% (11)
	• Fear of financial burden: 12% (6)
	• No fear: 14% (7)
Has the diagnosis of your tumor changed your daily routine or activities?	• Yes, significantly: 68% (34)
	• Yes, somewhat: 14% (7)
	• No, not at all: 14% (7)
	• I’m not sure: 4% (2)
Do you feel that people around you (family, friends, or others) understand what you’re going through?	• Yes, they understand fully: 74% (37)
	• Yes, they understand to some extent: 12% (6)
	• No, they don’t understand: 10% (5)
	• I am not sure: 4% (2)

On the financial front, 48% (24) felt a very high financial burden, and 20% (10) somewhat high. Support systems, assessed by asking patients about assistance from family, charitable organizations, or government programs, varied: 34% (17) reported strong support, while 44% (22) reported none. Regarding healthcare cost management, 40% (20) were confident, while 14% (7) were not, indicating varied coping strategies (Table-II).

**Table-II:** The Financial Impact of the Tumor on the Patient.

Question	Percentage (Frequency)
How would you describe the financial impact of your tumor diagnosis and treatment on your household?	• Very burdensome: 48% (24)
	• Somewhat burdensome: 20% (10)
	• Not very burdensome: 12% (6)
	• No impact at all: 20% (10)
Have you or your family had to make any significant financial sacrifices due to your tumor diagnosis or treatment?	• Yes, significant sacrifices: 30% (15)
	• Yes, some sacrifices: 14% (7)
	• No, there have been no sacrifices: 50% (25)
	• I am unsure: 6% (3)
Are you currently facing any of the following financial challenges due to your diagnosis? (Select all that apply)	• Difficulty paying for medical treatments: 28% (14)
	• Struggling to cover everyday living expenses (e.g., food, housing): 20% (10)
	• Difficulty accessing transportation for medical appointments: 6% (3)
	• Loss of income due to inability to work: 36% (18)
	• Increased out-of-pocket medical expenses (e.g., medications, hospital visits): 8% (4)
	• None: 36% (18)
Have you received any financial assistance or support (e.g., from family, charity organizations, or government programs) to help with medical costs?	• Yes, significant support: 34% (17)
	• Yes, some support: 18% (9)
	• No, I have not received any support: 44% (22)
	• I am not sure: 4% (2)
How has the financial burden affected your ability to access necessary treatments or follow-up care?	• It has significantly affected my access to treatment: 44% (22)
	• It has somewhat affected my access to treatment: 18% (9)
	• It has not affected my access to treatment: 36% (18)
	• I am unsure: 2% (1)
Do you feel that your financial situation influences your decisions regarding treatment options?	• Yes, it significantly influences my decisions: 60% (30)
	• Yes, it somewhat influences my decisions: 16% (8)
	• No, it does not influence my decisions: 22% (11)
	• I am not sure: 2% (1)
How confident are you that you will be able to manage your healthcare costs in the future?	• Very confident: 40% (20)
	• Somewhat confident: 42% (21)
	• Not confident: 14% (7)
	• I am unsure: 4% (2)
In your opinion, how much of a role do financial concerns play in your emotional well-being during your treatment?	• A significant role: 50% (25)
	• Somewhat of a role: 40% (20)
	• A minor role: 8% (4)
	• No role at all: 2% (1)

In terms of mental health symptoms, a substantial portion of patients (38%, 19) reported feeling tense most of the time, and another 30% (15) felt tense occasionally. The experience of feeling slowed down was reported nearly always by 34% (17) of patients. Furthermore, 36% (18) of patients reported that they hardly looked forward to things (Table-III).

**Table-III:** The Hospital Anxiety and Depression Scale (HADS).^8^

Question	Percentage (Frequency)
I feel tense or ‘wound up’	• Most of the time: 38% (19)
	• A lot of the time: 6% (3)
	• From time to time, occasionally: 36% (18)
	• Not at all: 20% (10)
I still enjoy the things I used to enjoy	• Definitely as much: 20% (10)
	• Not quite so much: 26% (13)
	• Only a little: 14% (7)
	• Hardly at all: 40% (20)
I get a sort of frightened feeling as if something awful is about to happen	• Very definitely and quite badly: 24% (12)
	• Yes, but not too badly: 20% (10)
	• A little, but it doesn’t worry me: 10% (5)
	• Not at all: 46% (23)
I can laugh and see the funny side of things	• As much as I always could: 38% (19)
	• Not quite so much now: 40% (20)
	• Definitely not so much now: 8% (4)
	• Not at all: 14% (7)
Worrying thoughts go through my mind	• A great deal of the time: 20% (10)
	• A lot of the time: 6% (3)
	• From time to time, but not too often: 34% (17)
	• Only occasionally: 40% (20)
I feel cheerful	• Not at all: 16% (8)
	• Not often: 12% (6)
	• Sometimes: 46% (23)
	• Most of the time: 26% (13)
I can sit at ease and feel relaxed	• Definitely: 40% (20)
	• Usually: 22% (11)
	• Not often: 20% (10)
	• Not at all: 18% (9)
I feel as though I am slowed down	• Nearly all the time: 34% (17)
	• Very often: 26% (13)
	• Sometimes: 28% (14)
	• Not at all: 12% (6)
I get a sort of frightened feeling like ‘butterflies’ in the stomach	• Not at all: 36% (18)
	• Occasionally: 44% (22)
	• Quite often: 8% (4)
	• Very Often: 12% (6)
I have lost interest in my appearance	• Definitely: 34% (17)
	• I don’t take as much care as I should: 34% (17)
	• I may not take quite as much care: 10% (5)
	• I take just as much care as ever: 22% (11)
I feel restless as I have to be on the move	• Very much indeed: 32% (16)
	• Quite a lot: 10% (5)
	• Not very much: 50% (25)
	• Not at all: 8% (4)
I look forward with enjoyment to things	• As much as I ever did: 22% (11)
	• Rather less than I used to: 26% (13)
	• Definitely less than I used to: 16% (8)
	• Hardly at all: 36% (18)
I get sudden feelings of panic	• Very often indeed: 16% (8)
	• Quite often: 24% (12)
	• Not very often: 34% (17)
	• Not at all: 26% (13)
I can enjoy a good book or radio or TV program	• Often: 20% (10)
	• Sometimes: 26% (13)
	• Not often: 6% (3)
	• Very seldom: 48% (24)

When asked about their future outlook, 66% (33) of patients did not feel discouraged about the future while 20% (10) reported feeling more discouraged. Notably, 90% (45) of patients reported no suicidal ideation, though 8% (4) experienced passive thoughts and 2% (1) wished to die (Table-IV).

**Table-IV:** The Beck Depression Inventory (BDI).^9^

Question	Percentage (Frequency)
How sad do you feel right now?	0 - I do not feel sad: 30% (15)
	1 - I feel sad much of the time: 46% (23)
	2 - I am sad all the time: 14% (7)
	3 - I am so sad or unhappy that I can’t stand it: 10% (5)
How hopeless or pessimistic do you feel about the future?	0 - I am not discouraged about the future: 66% (33)
	1 - I feel more discouraged about the future than I used to: 20% (10)
	2 - I have little hope for the future: 8% (4)
	3 - I feel hopeless about the future: 6% (3)
How do you feel about past achievements/failures?	0 - I do not feel like a failure: 62% (31)
	1 - I have failed more than I should have: 18% (9)
	2 - As I look back, I see a lot of failures: 16% (8)
	3 - I feel I am a complete failure as a person: 4% (2)
How much pleasure do you get from doing things?	0 - I get as much pleasure as I ever did from the things I enjoy: 58% (29)
	1 - I don’t enjoy things as much as I used to: 18% (9)
	2 - I get very little pleasure from the things I used to enjoy: 6% (3)
	3 - I can’t get any pleasure from the things I used to enjoy: 18% (9)
How guilty do you feel?	0 - I don’t feel particularly guilty: 76% (38)
	1 - I feel guilty over many things I have done or should have done: 18% (9)
	2 - I feel guilty most of the time: 2% (1)
	3 - I feel guilty all the time: 4% (2)
How deserving do you feel of punishment?	0 - I don’t feel I am being punished: 78% (39)
	1 - I feel I may be punished: 10% (5)
	2 - I expect to be punished: 4% (2)
	3 - I feel I am being punished: 8% (4)
How much do you dislike yourself?	0 - I feel good about myself: 74% (37)
	1 - I have some disliking of myself: 12% (6)
	2 - I dislike myself: 10% (5)
	3 - I hate myself: 4% (2)
How critical are you of yourself?	0 - I don’t criticize or blame myself more than usual: 70% (35)
	1 - I am more critical of myself than I used to be: 22% (11)
	2 - I criticize myself for all of my faults: 4% (2)
	3 - I blame myself for everything bad that happens: 4% (2)
How often do you think about death or suicide?	0 - I don’t have any thoughts of killing myself: 90% (45)
	1 - I have thoughts of killing myself, but I would not carry them out: 8% (4)
	2 - I would like to kill myself: 2% (1)
	3 - I would kill myself if I had the chance: 0% (0)
How often do you cry?	0 - I don’t cry any more than I used to: 52% (26)
	1 - I cry more than I used to: 34% (17)
	2 - I cry all the time now: 2% (1)
	3 - I used to cry but now I don’t cry anymore: 12% (6)
How restless or agitated do you feel?	0 - I am not particularly irritated or agitated: 34% (17)
	1 - I get frustrated or impatient a little more easily than I used to: 50% (25)
	2 - I feel irritated all the time: 14% (7)
	3 - I am always angry or mad: 2% (1)
How much interest do you have in people or activities?	0 - I have not lost interest in other people or activities: 34% (17)
	1 - I am less interested in other people or things than I used to be: 38% (19)
	2 - I have lost most of my interest in other people or things: 4% (2)
	3 - I have lost all of my interest in other people or things: 24% (12)
How difficult is it for you to make decisions?	0 - I can make decisions about as well as I ever could: 44% (22)
	1 - I have more difficulty in making decisions than I used to: 34% (17)
	2 - I have much greater difficulty in making decisions than I used to: 10% (5)
	3 - I can’t make decisions at all: 12% (6)
How worthless do you feel?	
	0 - I do not feel worthless: 70% (35)
	1 - I don’t consider myself as worthwhile and useful as I used to: 16% (8)
	2 - I feel more worthless as compared to others: 4% (2)
	3 - I feel utterly worthless: 10% (5)
How much energy do you have?	0 - I have as much energy as I ever did: 16% (8)
	1 - I have less energy than I used to have: 58% (29)
	2 - I don’t have enough energy to do much of anything: 12% (6)
	3 - I don’t have any energy at all: 14% (7)
How have your sleeping patterns changed?	0 - I have not experienced any change in my sleeping pattern: 30% (15)
	1 - I sleep somewhat more than usual: 32% (16)
	2 - I sleep somewhat less than usual: 22% (11)
	3 - I sleep much more than usual or much less than usual: 16% (8)
How irritable or easily angered are you?	0 - I am not more irritable than usual: 44% (22)
	1 - I am more irritable than I used to be: 42% (21)
	2 - I am much more irritable than I used to be: 6% (3)
	3 - I am irritable all the time: 8% (4)
How has your appetite changed?	0 - I have not experienced any change in my appetite: 40% (20)
	1 - My appetite is somewhat less than usual: 14% (7)
	2 - My appetite is much less than usual: 30% (15)
	3 - I have no appetite at all or I eat much more than usual: 16% (8)
How hard is it for you to concentrate or focus?	0 - I can concentrate as well as I ever could: 50% (25)
	1 - I have trouble concentrating on things, like reading the newspaper or watching TV: 30% (15)
	2 - I can’t concentrate on anything: 6% (3)
	3 - I can’t read, watch TV, or do anything because I can’t concentrate: 14% (7)
How tired or fatigued do you feel?	0 - I am no more tired than usual: 32% (16)
	1 - I get more tired than usual: 48% (24)
	2 - I am tired all the time: 12% (6)
	3 - I am too tired to do anything: 8% (4)
How much interest do you have in sex?	0 - I have not lost interest in sex: 58% (29)
	1 - I am less interested in sex than I used to be: 22% (11)
	2 - I have lost most of my interest in sex: 10% (5)
	3 - I have lost all of my interest in sex: 10% (5)

In terms of worry and irritability, 42% (21) of patients were not at all confident in controlling their worry. Irritability was also prevalent, with 44% (22) of patients reporting that they were not easily annoyed, while 20% (10) experienced irritability more than half the days, and 14% (7) were irritable nearly every day (Table-V).

**Table-V:** The Generalized Anxiety Disorder 7-item (GAD-7) Scale.^10^

Question	Percentage (Frequency)
Feeling nervous, anxious, or on edge	0: Not at all sure: 34% (17)
	1: Several days: 22% (11)
	2: Over half the days: 22% (11)
	3: Nearly every day: 22% (11)
Not being able to stop or control worrying	0: Not at all sure: 42% (21)
	1: Several days: 26% (13)
	2: Over half the days: 24% (12)
	3: Nearly every day: 8% (4)
Worrying too much about different things	0: Not at all sure: 30% (15)
	1: Several days: 26% (13)
	2: Over half the days: 14% (7)
	3: Nearly every day: 30% (15)
Trouble relaxing	0: Not at all sure: 48% (24)
	1: Several days: 16% (8)
	2: Over half the days: 18% (9)
	3: Nearly every day: 18% (9)
Being so restless that it’s hard to sit still	0: Not at all sure: 46% (23)
	1: Several days: 16% (8)
	2: Over half the days: 14% (7)
	3: Nearly every day: 24% (12)
Being easily annoyed or irritable	0: Not at all sure: 44% (22)
	1: Several days: 22% (11)
	2: Over half the days: 20% (10)
	3: Nearly every day: 14% (7)
Feeling afraid as if something awful might happen	0: Not at all sure: 48% (24)
	1: Several days: 26% (13)
	2: Over half the days: 8% (4)
	3: Nearly every day: 18% (9)

## DISCUSSION

This study investigates cognitive and emotional strain in brain tumor patients, focusing on the differential impacts of benign and malignant neoplasms in resource-limited settings. Although extensive research exists in Western countries, there is a notable gap in understanding these impacts in Low- and Middle-Income Countries (LMICs), particularly in Pakistan. Understanding these impacts in LMICs is crucial for developing appropriate support systems and interventions.

This study included 50 patients with a mean age of 36.72 (SD = 15.74) years. The cohort comprised 62% (31) males and 38% (19) females, differing significantly from Kasper et al.’s 2022 study, which reported a gender distribution of 66.7% (20) females and 33.3% (10) males, though with a higher mean age of 56.01 years.^11^ In terms of tumor classification, 66% (33) of our cohort had benign neoplasms, while 34% (17) had malignant diagnoses, differing from Pidani et al.’s 2020 study, which showed a more balanced ratio of 52% benign (69) and 48% malignant (63) cases.^4^ Tumor-specific analysis revealed meningiomas as the leading diagnosis in our population at 28% (14), followed by glioma at 14% (7), and pituitary adenoma and astrocytoma at 10% (5). These findings contrast with Shah et al.’s 2023 study, where gliomas represented the majority at 46.8% (117), followed by meningiomas at 21.2% (53).^12^

Regarding patients’ understanding of their tumor, 54% (27) reported complete understanding, while 22% (11) indicated somewhat clear understanding. Anxiety and fear were the major emotions affecting 28% (14) and 34% (17) of patients, respectively. In Goebel et al.’s 2011 study, however, patients reported higher rates of worry (46%), fear (48%), and sadness (32%).^13^ Moreover, 84% (42) of our cohort expressed strong faith in their proposed treatment plan. This difference in emotional response patterns suggests that modern approaches to patient communication and support that were not implemented in this study may be more effective in managing psychological distress while maintaining high treatment confidence. The lower rates of negative emotions in our cohort, despite similar diagnostic contexts, indicate potential improvements in contemporary healthcare practices focused on patient education and psychological support.^14^

Financial burden was a significant challenge for many patients, with 48% (24) reporting high financial burden and 20% (10) experiencing moderate burden. Additionally, 52% (26) of patients received some form of financial support. The financial impact extended beyond this, with 60% (30) indicating that financial considerations significantly influenced their treatment decisions. Treatment costs varied considerably, with Pidani et al. (2020) documenting that 34% (45) of patients paid between 200,000-800,000 Pakistani Rupees (PKR), 36% (47) paid between 800,000-1,200,000 PKR, and 30% (40) spent over 1,200,000 PKR.^4^ These costs represented substantial financial strain, considering Pakistan’s average monthly income of 41,545 PKR in 2019.^15^ However, patients remained relatively optimistic about future healthcare costs, with 40% (20) expressing strong confidence and 42% (21) indicating moderate confidence in their ability to manage future expenses.

The Hospital Anxiety and Depression Scale (HADS) was used to assess emotional health. Our findings indicated that 34% (17) of patients exhibited mild to clinically relevant anxiety and depression, closely aligning with Keeling et al.’s 2013 study, which reported an identical prevalence rate of 34%.^16^ In contrast, Kasper et al.’s 2022 study documented a higher prevalence of 40% among participants scoring above the threshold of 7.^11^,^12^ Bunevicius et al.’s 2013 study revealed mild psychological distress in 33% (14) of patients and severe distress in 43% (18).^17^

The Beck Depression Inventory (BDI) was used to assess depression in patients. Most patient responses indicated low levels of severe depressive symptoms, with 66% (33) expressing no discouragement regarding their future prospects. Suicidal ideation was absent in 90% (45) of cases, while 76% (38) reported minimal feelings of guilt. These findings are consistent with broader research, though methodological variations exist. For example, Bunevicius et al.’s 2013 study identified mild-to-severe depressive symptoms (threshold ≥8) in 45% (22) of patients, whereas Hickmann et al.’s 2016 investigation found such symptoms (threshold ≥11) in 25.5% of assessments conducted over nine months.^5^,^17^

According to the GAD-7 assessment results, approximately half of the patients exhibited persistent anxiety-related symptoms. Specifically, 22% (11) reported daily feelings of nervousness and anxiety, while 30% (15) experienced daily worries. Additional symptoms included restlessness in 24% (12) and difficulty relaxing in 18% (9). Fehrenbach et al.’s 2021 study categorized GAD-7 scores into four levels (minimal: 0-4, mild: 5-9, moderate: 10-14, severe: 15-21) and used a cut-off value of ≥10 to identify significant anxiety, with a sensitivity of 89% and specificity of 82%.^18^ In our cohort, 77.4% (24) demonstrated minimal levels of anxiety while 22.6% (7) showed significant levels of anxiety. However, a longitudinal assessment conducted over nine months revealed that 25.5% of evaluations had scores ≥11, indicating mild depressive symptoms.^18^

### Strengths and Limitations:

This study utilized a pre-validated questionnaire, ensuring reliability in data collection. Additionally, the translated questionnaire was verified by a language expert to ensure accuracy and cultural appropriateness. All interviews were conducted with the informed consent of both the patients and the data collectors, and voice recordings of the interviews were included to maintain full transparency.

Although the questionnaire was translated into the national language, communication barriers persisted, as many patients were more fluent in regional languages such as Punjabi or Saraiki, and the questionnaire was not multilingual. A significant limitation was the small sample size of 50 participants, which was insufficient for the originally intended nationwide study. Due to logistical constraints, data were only collected from two cities-Lahore and Islamabad-thus limiting the generalizability of the findings to other regions, making it a regional study rather than a national one. Additionally, patients may have been affected by interviewer bias due to the awareness of being recorded, which could have led them to alter their responses in ways they felt were more acceptable. The length of the questionnaire also contributed to participant fatigue, leading to reduced attention during the interview process.

### Clinical Recommendations:

Psychological screening and cognitive assessments should be routinely conducted for patients diagnosed with brain tumors. The integration of psycho-oncological care alongside conventional cancer treatments should be prioritized to address the psychological and emotional needs of these patients. Counseling services focused on helping patients understand their diagnosis are crucial in reducing anxiety and improving adherence to treatment protocols. Additionally, the implementation of interventions such as mindfulness and cognitive rehabilitation programs should be considered to further alleviate emotional distress. In resource-limited settings, it is essential to provide structured financial support as part of comprehensive patient care to mitigate the financial burden on patients.

## CONCLUSION

Whether benign or malignant, brain tumors pose a significant challenge for patients in Pakistan and globally. These tumors impact patients psychologically, financially, and socially, often leading to anxiety and depression, even when patients are confident in their treatment plans. Addressing the mental health needs of cancer patients in Pakistan is crucial for improving their quality of life, treatment adherence, and reducing the stigma surrounding mental health symptoms. Further research with a larger, more diverse sample is necessary to gain a deeper understanding of these challenges in Pakistan and guide the development of targeted interventions.

### Authors’ Contribution:

**MB:** Data analysis and interpretation, critical review of manuscript.

**MR, AA, SR, OBA:** Data acquisition and drafting the manuscript.

**HMQ:** Concept and design of the study, critical review of manuscript and supervision.

All authors have approved the final version and are accountable to the integrity of the study.
